# How long do patients with chronic disease expect to live? A systematic review of the literature

**DOI:** 10.1136/bmjopen-2016-012248

**Published:** 2016-12-21

**Authors:** Barnaby Hole, Joseph Salem

**Affiliations:** 1 Department of Renal Medicine, Southmead Hospital, Bristol, UK; 2 Department of Medicine, University of Bristol, Bristol, UK

**Keywords:** chronic disease, prognosis, life expectancy, chronic kidney disease

## Abstract

**Objective:**

To systematically identify and summarise the literature on perceived life expectancy among individuals with non-cancer chronic disease.

**Setting:**

Published and grey literature up to and including September 2016 where adults with non-cancer chronic disease were asked to estimate their own life expectancy.

**Participants:**

From 6837 screened titles, 9 articles were identified that met prespecified criteria for inclusion. Studies came from the UK, Netherlands and USA. A total of 729 participants were included (heart failure (HF) 573; chronic obstructive pulmonary disease (COPD) 89; end-stage renal failure 62; chronic kidney disease (CKD) 5). No papers reporting on other lung diseases, neurodegenerative disease or cirrhosis were found.

**Primary and secondary outcome measures:**

All measures of self-estimated life expectancy were accepted. Self-estimated life expectancy was compared, where available, with observed survival, physician-estimated life expectancy and model-estimated life expectancy. Meta-analysis was not conducted due to the heterogeneity of the patient groups and study methodologies.

**Results:**

Among patients with HF, median self-estimated life expectancy was 40% longer than predicted by a validated model. Outpatients receiving haemodialysis were more optimistic about prognosis than their nephrologists and overestimated their chances of surviving 5 years. Patients with HF and COPD were approximately three times more likely to die in the next year than they predicted. Data available for patients with CKD were of insufficient quality to draw conclusions.

**Conclusions:**

Individuals with chronic disease may have unrealistically optimistic expectations of their prognosis. More research is needed to understand how perceived life expectancy affects behaviour. Meanwhile, clinicians should attempt to identify each patient's prognostic preferences and provide information in a way that they can understand and use to inform their decisions.

**Trial registration number:**

CRD42015020732.

**Strengths and limitations of this study**

- This is the first review of perceived life expectancy among patients with chronic non-cancer disease.
- The findings build on and reproduce the oncology literature showing patients with cancer have a tendency to overestimate their life expectancy and chances of cure.
- The findings of this review are based on the small number of studies that have been conducted on this subject.
- The literature was only available for patients with heart failure, end-stage renal failure and chronic obstructive pulmonary disease.

## Introduction

How long an individual expects to live—their perceived life expectancy—reflects their disease understanding and the medical profession's ability to prognosticate for and communicate with them. Perceived life expectancy may affect a variety of outcomes, including healthcare choices. Patients with incurable lung and colon cancer who thought they were going to live for at least 6 months were more likely to favour life-extending therapy over comfort care compared with patients who thought there was at least a 10% chance that they would not live 6 months.^1^ Critically unwell inpatients who do not expect to live 2 months are less likely to opt for cardiopulmonary resuscitation in the event of sudden death than individuals who perceive their prognosis to be better.^2^

Prognosis communication has been widely studied in oncology, and the majority of people with cancer want detailed prognostic information, presented honestly and openly.^3^ However, non-cancer chronic disease causes more deaths than cancer worldwide, with cardiovascular disease being the biggest killer.^4^ Almost 2.3 million people in the UK have a diagnosis of coronary heart disease, and over half a million have heart failure (HF).^5^ An estimated 1.2 million people have a diagnosis of chronic obstructive pulmonary disease (COPD)^6^ and almost 60 000 receive renal replacement therapy for end-stage renal failure (ESRF).^7^ Life expectancy for patients with chronic disease including advanced COPD, HF and ESRF can be as poor as that seen in incurable cancer.^8–10^

Lately, there has been a practice shift away from paternalistic medicine. Shared decision-making empowers individuals and their carers to make choices about what care they want based on honest, open disclosure of the known benefits and risks of proposed treatment options.^11^ Decisions to accept treatment with invasive therapies such as ventilation, dialysis and implanted cardiac defibrillator placement may be influenced by how long individuals expect to live. Patients facing such decisions can only be considered fully informed if they have an understanding of their prognosis and the effects available treatments might have on it. Up to 38% of patients near the end of life receive treatment administered with little or no hope of it having any effect, largely because of the underlying state of the patient's health and the known or expected poor prognosis regardless of treatment.^12^ Quality of end-of-life care is significantly better for patients with cancer than for patients with ESRF or HF, largely due to higher rates of palliative care review and lower rates of intensive care admission and cardiopulmonary resuscitation among individuals with malignancy.^13^ It is possible that suboptimal end of life treatment is partly driven by unrealistic expectations of prognosis.

Many patients with cancer, including those with incurable disease, report never discussing prognosis with their healthcare team, misunderstand whether their condition is curable and overestimate their expected survival.^3^ No systematic analysis of perceived life expectancy among individuals with non-cancer chronic disease has been performed. This review was conducted to evaluate what is known about how long patients with non-cancer chronic disease expect to live and how these estimates compare with other methods of predicting survival and measured outcomes.

## Methods

### Search strategy

A systematic search of MEDLINE, Embase, PsychINFO and the Cochrane Library was conducted up to and including September 2016. Abstracts of unpublished works were searched using ProQuest dissertations and theses search and the Networked Digital Library of Theses and Dissertations Global ETD search. Search terms relating to ‘life expectancy’ and ‘self-estimated’ were used (see online supplementary appendix A). Search results were limited to humans and English language.

10.1136/bmjopen-2016-012248.supp1 supplementary appendix

### Inclusion and exclusion criteria

Non-cancer chronic disease was defined as any long-term illness that is associated with reduced life expectancy, but not caused by cancer or infection. Conditions included were HF; chronic kidney disease stage 5 (CKD); ESRF receiving dialysis or conservative care; diabetes mellitus; COPD; interstitial lung disease; neurodegenerative disease and liver cirrhosis. Studies were included where adults (≥18 years of age) with these conditions were asked to estimate their life expectancy. All measurements of life expectancy were accepted, including those in terms of duration (eg, “How long do you expect to live”), and chance (eg, “What is the chance you will be alive in five years”). Studies were excluded where only self-estimated probability of ‘cure’ was determined, where the only option for survival duration was <6 months and where participants were asked to consider only hypothetical situations (eg, “How long do you think you would live if you had a kidney transplant”). Studies reporting only on participants with cancer, HIV/AIDS, congenital heart disease, cystic fibrosis and organ transplant were excluded. In all these conditions the situation, illness culture or advances in treatment may have affected how generalisable findings were to the larger chronic disease population. At the title and abstract searching phase, articles assessing prognosis in excluded diagnoses were not rejected, so that reference list searches could be performed from these papers. Where studies reported a mixture of included and excluded diagnoses, they were incorporated if the data on individual diseases were reported separately. Where data were not separately reported, authors were contacted to request online supplementary files. Data were extracted from figures and tables in papers, where needed.

### Study selection process

Titles were independently examined by two reviewers (BH and JS) according to the above criteria and a Kappa statistic calculated to assess agreement. Abstracts from titles accepted by either one or both reviewers were collected and assessed independently, using the same criteria, and included if both recommended inclusion. Where only one reviewer recommended inclusion, a consensus decision was made after discussion. Full text articles were requested and read and reference lists were examined with additional papers included by the same criteria. At this point, papers reporting excluded disease groups were rejected. Disagreement between authors was addressed by discussion and a consensus decision reached in all cases.

### Quality assessment

No suitable tool to grade the quality of included literature could be found. A quality assessment tool (see online supplementary appendix B) was developed by the authors to assess and grade the quality of available literature based on semiobjective assessment of factors influencing the generalisability, risk of bias and reporting quality of included literature. This tool has not been previously validated. Papers included for review were independently graded by the authors and a mean score taken to categorise each as low, medium or high quality. The study was registered with the PROSPERO database, registration number CRD42015020732.

10.1136/bmjopen-2016-012248.supp2 supplementary appendix

## Results

**Figure 1:**
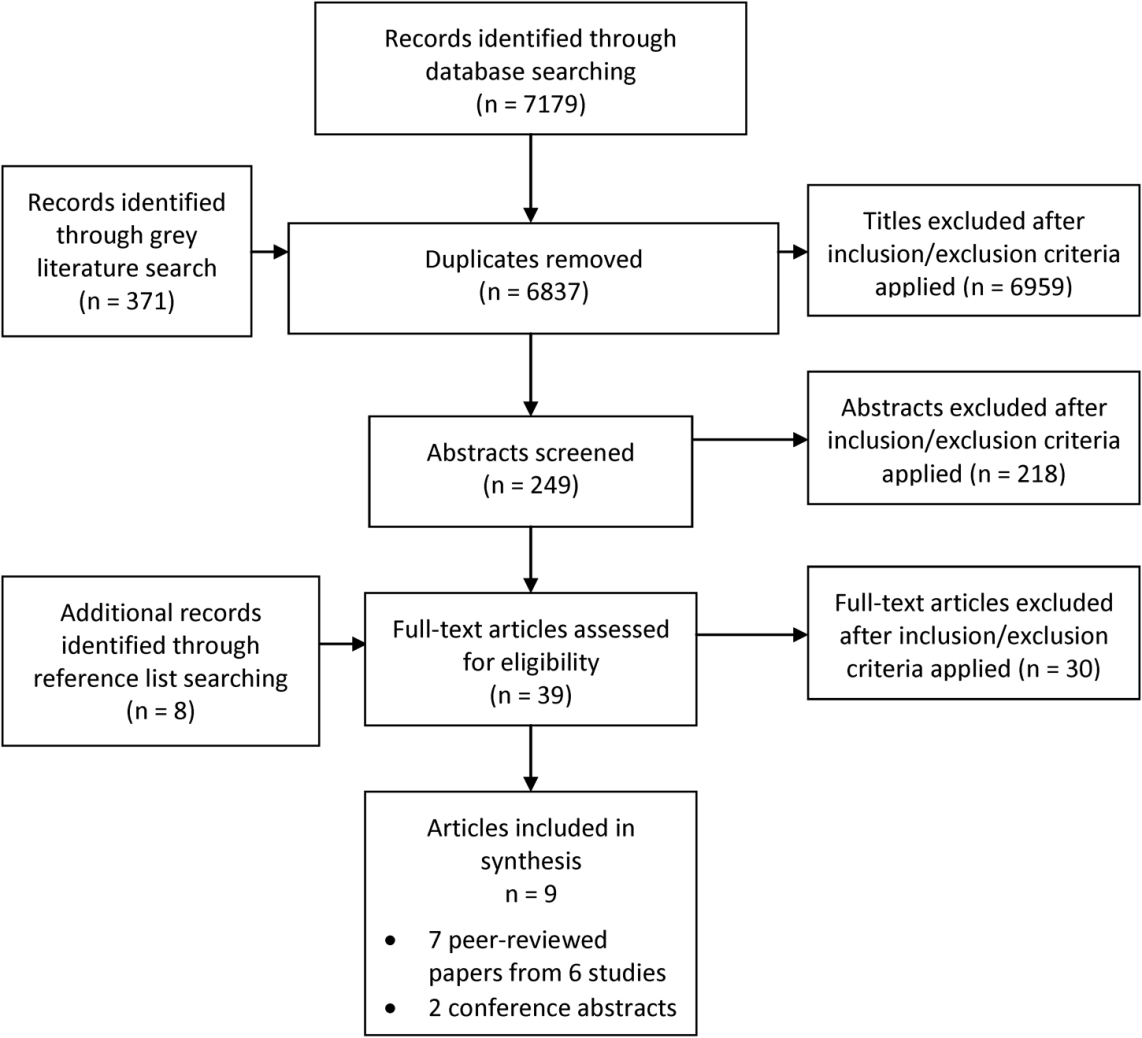
PRISMA diagram.

The initial search provided 6837 titles after removal of duplicates. 249 abstracts were selected for review by either one or both authors (agree to exclude, 6588; agree include, 158; disagree, 91; κ 0.77). Thirty-one articles were collected, and reference list searching provided an additional eight. After full text examination of 39 articles, seven papers and two conference abstracts were included in the review (figure 1). No unpublished works met the inclusion criteria. Two of the included papers originate from a single study.^14^ ^15^ A complete list of papers including reasons for inclusion/rejection is available (see online supplementary appendix C). Evidence was graded as medium quality in four and low quality in three of the included papers (table 1). No articles were graded as high quality. The two abstracts were not quality assessed as insufficient information was available.

**Table 1:** Summary of included articles

Reference	Conditions	Origin	Quality	Design	Patients included	Measures used	Results	Summary	Pros + and cons −
Allen *et al*^16^ 2008	HF	USA	Medium	Cross-sectional interviewer-administered questionnaire in a single centre outpatient heart-failure service	122 sequentially recruited participants with HF (NYHAI-IV) Mean age 61 (IQR 53–74) 62% male 47% African-American	Patients were asked “If you had to guess, how much longer do you think you will live?” and completed Multichoice answers ranging from <3 months to >10 years, and A visual Analogue Scale, marking their estimated age at death Model-predicted life expectancy using the Seattle Heart Failure Model Observed survival over median follow-up of 3 years	Median self-estimated life expectancy was 13 years (IQR 8–21; range 1–54 years) Median model-predicted life expectancy was 10 years (IQR 7.2–13.3; range 2.0–25 years) 66% of patients overestimated their survival compared with the model by 30% or more The median overestimate was 40% 29% of patients died within 3 years	Self-estimated-life expectancy was on average significantly greater than that predicted by a validated model Younger age, greater disease severity and measures of less depression were independently associated with overestimation of survival	+ Efforts made to improve and check patient understanding of question − 26 of 148 enrolled participants felt unable/unwilling to estimate survival − Only 35 of 122 patients were followed up until their death − Only 9 of 122 patients had NYHA IV HF − No index group without chronic disease was included
Fried *et al*^15^ 2003	COPD HF	USA	Medium	Cross-sectional interview survey administered to patients registered at community practices and outpatient clinics of two hospitals, and inpatients of three hospitals. **Same patient group as Fried *et al* 2006**	135 patients with COPD or HF, aged 60 and older, meeting criteria for limited life expectancy and requiring assistance with daily living COPD—79 patients Mean age 72 (SD 7) 51% Male 92% White HF—56 patients Mean age 75 (SD 8) 70% Male 88% White	Patients and clinicians were asked how long they thought the patient would live and answered using multichoice options ranging from <1 month to >10 years	Only 9 of 135 patients expected to live <1 year, but 38 patients died over this period. 58 of 79 patients who responded to being asked to estimate their own life expectancy expected to live 2 years or more Of the 65 available patient–clinician pairs who both responded, 34 agreed the prognosis was 2 years or more, 9 agreed the prognosis was 2 years or less, 7 clinicians thought the patient would live 2 years or more when the patient did not expect to live this long and 15 patients expected to live 2 years or more when their clinician was less optimistic Kappa was 0.22 suggesting very poor agreement	Patient expectations of 1 year mortality are higher than observed. Agreement between patients and their clinicians about likely prognosis is poor.	− 56 of 135 patients were unable or unwilling to estimate their life expectancy − No index group without chronic disease was included
Fried *et al*^14^ 2006	COPD HF	USA	Medium	Serial interview survey administered to patients registered at community practices and outpatient clinics of two hospitals, and inpatients of three hospitals. **Same patient group as *Fried et al* 2003**	135 patients with COPD or HF, aged 60 and older, meeting criteria for limited life expectancy and requiring assistance with daily living COPD—79 patients Mean age 72 (SD 7) 51% Male 92% White HF—56 patients Mean age 75 (SD 8) 70% Male 88% White	Patients were asked how long they thought the patient would live and answered using multichoice options ranging from <1 month to >10 years	9 of 59 patients who responded expected to live <1 year at their first interview. Of 59, 5 expected to live <1 year at their final interview 38 of 135 patients died over this period	Patient expectations of 1 year mortality are higher than observed The majority of patients (those who were alive and dead at the end of the year-long study) made no adjustment to their self-estimated life expectancy	− 56 of 135 patients were unable or unwilling to estimate their life expectancy − No index group without chronic disease was included
Kraai *et al*^17^ 2013	HF	The Netherlands	Low	Cross-sectional questionnaire administered in outpatient setting in one HF clinic. Subcomponent of time trade-off study	100 patients with HF (NYHA I–IV) all over 50 years of age. Mean age 70 (SD 9.4) 71% male	Visual Analogue Scale from 50 to 100 years of age; patients were asked to indicate the most accurate estimation of their life expectancy	Mean life expectancy indicated by patients was 82 (SD 8.6) years. No difference in self-estimated life expectancy was found between patients unwilling vs willing to trade time	Self-estimated life expectancy probably exceeds likely outcomes, but no comparator data was available. Despite patients with more advanced or symptomatic HF being more willing to trade time, no difference was found between the groups in terms of expected longevity	− No comparator prediction or measurement of survival used −Only 2 of 100 patients had NYHA IV HF − No index group without chronic disease was included
Shah *et al*^18^ 2006	HF COPD CKD	UK	Low	Cross-sectional interviewer-administered questionnaire in outpatient and inpatient settings at one acute NHS Trust and a neighbouring hospice	20 patients in total meeting criteria for limited life expectancy:6 HF (NYHA III/IV) 9 COPD 5 CKD Median age 72 50% male 85% white	Patients and physicians chose one of seven short prognosis statements that most accurately predicted how their illness might affect their life expectancy	7 of 20 (35%) patients estimated their prognosis to be <1 year 13/17 physicians (76%) estimated their patient's prognosis to be < 1 year	Exploratory study, no firm conclusions available	− Very small numbers − Sample poorly representative of a general outpatient population − No index group without chronic disease was included
Stewart *et al*^19^ 2010	HF	USA	Low	Cross-sectional written questionnaire with inpatients and outpatients from two HF centres. Subcomponent of time trade-off study.	105 patients with LVEF <35% and symptomatic HF Mean age 58 (SD 13) 70% male	Methodology for collecting self-estimated life expectancy not described	65% thought they would live more than 10 years and 34% believed they would be alive for at least 20 years. Patients willing to trade more time expected shorter survival than those unwilling to trade time. 46% of the patients willing to trade away at least 12 months anticipated that they would not survive 5 years. No difference was found in self-estimated survival between inpatients and outpatients (data not provided)	Self-estimated life expectancy probably exceeds likely outcomes, but no comparator data was available. Willingness to trade time is associated with shorter self-estimated life expectancy	− No comparator prediction or measurement of survival − Only 3 of 105 patients had NYHA IV HF − Study methodology and tool not described − No index group without chronic disease was included
Wachterman *et al*^20^ *2013*	ESRF	USA	Medium	Cross-sectional interviewer-administered questionnaire in two community-based haemodialysis units.	62 patients receiving maintenance haemodialysis with 20% or greater predicted risk of dying in the next year. Mean age 68 (SD 10) 42% Male 52% Black	Patients asked what they thought their chance was of being alive at 1 and 5 years (≥90%, about 75%, about 50%, about 25%, ≤10%, don't know). Nephrologist in charge of care asked to estimate each patients’ chance of being alive at 1 and 5 years on a continuous scale of 0% to 100%. Survival data with follow-up of 23 months	For 1 year survival prediction, patients were more optimistic in 64% of patient–nephrologist pairs, whereas nephrologists were more optimistic in only 10%. For 5 year survival prediction, patients were more optimistic in 69% patient–nephrologist pairs, whereas nephrologists were more optimistic in only 2% Only 6% of patients thought they had a <50% chance of being alive at 5 years, whereas actual survival at 23 months was only 56%	Patient expectations of 5-year mortality are higher than observed. Patients were significantly more optimistic about their survival than their nephrologists. Patients’ 1 year survival expectations were more consistent with actual survival than clinician estimates. Patients who expected to live longer were more likely to opt for life-extending treatments	− 88 of 150 eligible patients were excluded or refused to participate − No index group without chronic disease was included
Ambardekar *et al*^21^ 2016 (abstract only)	HF	USA	Not rated	Cross-sectional report of self-estimated life expectancy. Methodology not reported. Subcomponent of multicentre prospective cohort study	161 ambulatory patients with advanced HF from 10 American centres	Patient self-assessment of life expectancy Outcomes at mean follow-up of 13 months Methodology for data collection not described	64% of patients identified by a physician to have ‘high-risk’ HF estimated a life expectancy of >2 years. 40% died, were transplanted or required a mechanical left-ventricular assist device over a mean follow-up of 13 months	Patients expectations of outcome were optimistic compared with physician-predicted or observed outcomes	+ Multicentre prospective cohort − Abstract only at time of review − No index group without chronic disease was included
O'Donnell *et al*^22^ 2015 (abstract only)	HF	USA	Not rated	Self-assessment of prognosis in single centre cohort of hospitalised patients with HF. Methodology incompletely reported	23 participants Mean age 73 66% Male 77% White	Patient self-assessment of life expectancy	70% of patients estimated a life expectancy of >5 years 43% of patients estimated a life expectancy of >10 years	Self-estimated life expectancy probably exceeds likely outcomes, but no comparator data were available. Patients who did not want to discuss prognosis all expected to live >10 years	− Very small numbers − Abstract only at time of review

CKD, Chronic kidney disease; COPD, chronic obstructive pulmonary disease; ESRF, end-stage renal failure; HF, heart failure; LVEF, left ventricular ejection fraction.

10.1136/bmjopen-2016-012248.supp3 supplementary appendix

Studies came from the UK,^18^ Netherlands^17^ and USA.^14^ ^16^ ^19–22^ A total of 729 participants were included (HF, 573; COPD, 89; ESRF, 62; CKD, 5) with study sizes ranging from 20 to 135 patients (see table 1). Four papers reported on a single medical disease; HF^16^ ^17^ ^19^ ^21^ ^22^ and ESRF.^20^ Others reported on a mixture of conditions; HF and COPD^14^ ^15^ and HF, CKD and COPD.^18^ No papers reporting on non-COPD lung disease, neurodegenerative disease or cirrhosis were found.

The mean age of study participants ranged from 58 to 75. In the study by Fried *et al*^14^ ^15^ only individuals over 60 years of age were recruited and only those over 50 in the study by Kraai *et al.*^17^ No minimum age was set in the other studies. Two studies did not include selection criteria for disease severity,^16^ ^17^ and selection criteria were unreported in one study.^21^ In all other studies, criteria were used to select for patients with advanced disease. Patients with ESRF were all receiving outpatient haemodialysis.^20^ Reported levels of comorbidity were high. The mean Charlson Comorbidity Index for patients with ESRF was 5.8 (SD 1.6).^20^ Among US patients with HF in one study, 82% had hypertension, 54% diabetes and 29% COPD.^16^ Among patients with HF from the Netherlands, 57% had hypertension, 30% had diabetes, 24% had COPD and 11% had a stroke.^17^

One study used a written questionnaire to measure self-estimated life expectancy.^19^ Methodology was unreported in two studies.^21^ ^22^ All other studies used interviews. Participants with ESRF were asked about their chances of being alive at different time points.^20^ In the other studies, participants were asked to indicate how long they expected to live by selecting from vignette answers,^18^ giving a verbal response^14–16^ and/or by using a Visual Analogue Scale.^16^ ^17^ In one study, it was not possible to ascertain how the question had been posed or answered.^19^ For studies where data were available, large numbers of initially eligible patients were excluded from the studies, largely on the grounds of language skills or cognitive impairment (range: 88/150 (59%);^20^ 82/238 (34%);^17^ 82/361 (23%);^14^ ^15^ 4/44 (9%))^18^. Some participants were unable or unwilling to provide a self-estimate of life expectancy (range: 56/135 (41%);^14^ ^15^ 26/148 (18%);^16^ 3/62 (5%);^14^ ^15^ ^20^ 0/40 (0%)).^18^

Self-estimates of life expectancy were compared with predictions from clinical risk calculators,^16^ clinician-estimated life expectancy,^14^ ^15^ ^18^ ^20^ observed survival^14–16^ ^18^ ^20^ ^21^ or presented without comparator data.^17^ ^19^ ^22^ Follow-up periods ranged from 1 to 3 years, and the majority of patients (range 56–73%) were alive at the end of the studies. Analysis was performed in one study to characterise factors associated with overestimation of survival.^16^ In three papers, patients were asked about their preferences around treatment aims, and analyses performed looking at how these responses correlated with self-estimated life expectancy.^17^ ^19^ ^20^ One paper used repeat measures to examine how self-estimated life expectancy changed with disease course.^14^

### Self-estimated life expectancy compared with observed survival

Comparisons of self-estimated life expectancy and observed survival were reported in five papers from four studies^14–16^ ^18^ ^20^ and one abstract.^21^ In general, self-estimated life expectancy exceeded observed survival. The only example of self-estimated life expectancy consistent with survival was 1-year mortality in patients with ESRF.^20^ 81% of patients thought they had a better than 90% chance of being alive at 1 year. Observed survival was 93%. In comparison, 96% of patients believed they had a better than 50% chance of being alive at 5 years, but 44% had died within just 23 months. In one study, only 5% of patients with HF estimated their life expectancy to be 3 years or less, but observed mortality was 29% after a median follow-up of 3.1 years.^16^ Among patients with advanced HF, 3 of 56 (5%) patients expected to live <1 year, but 17 (30%) were dead in this period.^15^ Furthermore, 6 of 79 (8%) patients with COPD in the same study predicted their life expectancy to be <1 year; 21 (27%) died. When interviewed within the 90 days before they died, only 2 of 16 patients predicted their life expectancy to be less than a year.^14^ In the study published only as an abstract, 64% of patients with HF expected to live for longer than 2 years, but at a mean follow-up of 13 months 40% had died, been transplanted or required a left-ventricular assist device.^21^ Patient numbers were too low in one study to draw conclusions from observed survival.^18^

### Self-estimated life expectancy compared with model predictions of survival

In the only study that used a validated model^23^ to predict survival, self-estimated life expectancy exceeded model predictions.^16^ The median self-estimated life expectancy for 122 patients with HF was 13 years and the median model-predicted life expectancy was 10 years. There was no significant relationship between self and model-predicted life expectancy. The median ratio between self-estimated and model-estimated life expectancy was 1.4; indicating a 40% overestimation. Self-estimates of life expectancy were more similar to model predictions based on age and gender alone than to predictions taking heart disease into account.

### Self-estimated life expectancy compared with clinician-estimated life expectancy

Four papers from three studies reported comparisons of self-estimated and clinician-estimated life expectancy.^14^ ^15^ ^18^ ^20^ Estimates agreed poorly, with a tendency for patients to be more optimistic about life expectancy than their clinicians. Estimating 1-year and 5-year survival, patients with ESRF on dialysis were significantly more optimistic than their nephrologists.^20^ Among patients with COPD and HF, agreement between patients and their clinicians about whether the patient would survive 2 years was poor, with a Kappa statistic of 0.22.^15^ Numbers of patients in one study were too small for any conclusions to be drawn.^18^

### Other findings

Younger age, greater disease severity and lower levels of depression were independently associated with self-estimated life expectancy exceeding model predictions among patients with HF.^16^ Patients receiving haemodialysis who thought they had a ≥90% chance of being alive in 1 year were significantly more likely to choose life-extending therapy (44%) than patients who reported a <90% chance (9%).^20^ Patients with advanced COPD and HF serially interviewed over 1 year showed no evidence of adjusting their self-estimated life expectancy with disease progression.^14^ Only one patient of 135 revised their estimate from >1 year to <1 year, while mortality was 28% over this period. Three studies found that patients with HF make estimates of their life expectancy that are likely to be optimistic but did not present any other validated prediction or measure of survival.^17^ ^19^ ^22^ One found patients who anticipated shorter survival to be more willing to trade longevity for improved quality of life than those who predicted longer lives.^19^ The other study did not demonstrate this.^17^ One study was published only as an abstract and had insufficient numbers of patients to draw conclusions.^22^

## Discussion

Practice guidelines advocate considering prognosis when making decisions with patients who have chronic disease^24^ ^25^ and promote sharing survival statistics with patients.^26^ ^27^ There is evidence from cancer^14^ ^28^ ^29^ and non-cancer^15^ ^30^ ^31^ literature that patients with life-limiting illness want open and honest communication about their prognosis. Where treatment options differ markedly in survival benefit, patients require an understanding of their life expectancy with each treatment to make fully informed decisions between them. Hospitalised individuals are more likely to want cardiopulmonary resuscitation if they expect to survive their illness, even if these expectations are improbable.^2^ ^32^ Patients with terminal cancer who are optimistic about their prognosis are more interventional in their choice of medical therapy.^1^ It is conceivable that behaviours as diverse as adherence to preventative drugs and deciding whether to make a will could be influenced by how long an individual expects to live.

In this systematic review of self-estimated life expectancy in chronic disease, individuals' estimates exceeded nearly all predictions and measures of survival; including model-predicted and observed survival. Patients with non-cancer chronic disease may have survival expectations that markedly exceed outcomes. These expectations might lead some patients to make health decisions and life choices that they would not if their predictions were more realistic. Patients were more optimistic than their clinicians when estimating life expectancy. Only in one instance (1 year survival in ESRF) were patients' estimations in keeping with actual survival, and more accurate than their physicians', but by 2 years this had reversed.^20^ Whether this time-based effect represents a reproducible feature of perceived versus clinician-predicted life expectancy would require replication in other disease groups. Patients with HF and COPD were approximately three times more likely to be dead within the year than they predicted.^15^ Life expectancy was overestimated by a median of 40% by patients with HF, when compared with a validated model; equating to 3 years of life for the average patient.^16^ Self-estimates were more in keeping with the life expectancy of matched adults without chronic disease.^16^ There was evidence that no meaningful adjustment in expected survival is made by patients approaching the ends of their lives.^14^

If the findings of this review reflect pervasive overestimation of life expectancy by individuals with chronic disease, there are several possible explanations. First, patients might never be informed that their condition could affect their life expectancy. Such individuals are likely to base survival expectations on familial and media exposure, influenced by hopefulness and ‘fighting spirit’. Others might receive overoptimistic forecasts; either due to methods of estimation, or adjustment by the communicating clinician. Finally, patients might be provided with appropriate quantitative estimates, but instead form more favourable personal predictions.

These findings are compatible with the oncology literature. Most patients with cancer want to discuss life expectancy, although desire for quantitative estimation varies.^33^ Despite this, many report not having discussed prognosis or are found to misunderstand the status of their disease, the aim of their treatment and their prognosis.^3^ Overestimation of the chances of cure and survival is common, even if disease is incurable and where individuals report having discussed prognosis with their clinician.^34^ The prognosis in non-cancer disease can be equivalently poor to that seen in malignancy.^8–10^ End of life care differs by diagnosis, so caution must be taken when generalising findings from cancer to non-cancer disease settings.^13^ ^35^

None of the patients with ESRF in this review recalled discussing life expectancy with their clinician; their nephrologists reported having such conversations with only 3% of the patients.^20^ Sixty-three per cent of patients with HF in one study did not recall having spoken with their physician about their prognosis following the diagnosis of HF and only 36% believed HF would shorten their life.^16^ Only 22% of patients in one study with advanced COPD and HF recalled having been told that they could die of their disease and only 1% recalled having been given an estimate of how long they might live.^15^ Prognostic discussions between patients with non-cancer chronic disease and their clinicians may be infrequent. In a systematic review of the literature, it was found that most patients with COPD report that they have never had an end of life care discussion with a healthcare provider.^36^ Interviews with individuals with ESRF suggest that while early information is beneficial, the daily focus on clinical care and a reliance on clinicians to initiate end of life care discussions act as barriers to advance planning.^31^ Interviews with patients with ESRF and their clinicians suggest that nephrologists tend to avoid discussions about the future.^37^ The evidence for prognostic discussions between patients with cancer and their clinicians is varied.^3^ Discussions are more likely to be triggered by the clinician than the patient and are probably infrequent among individuals with advanced malignancy.^3^ Where discussions occur, they are often unclear and both parties tend to avoid acknowledging or discussing prognosis.^38^ There are boundaries to clinicians initiating prognostic discussions, such as fear of causing anxiety or destroying hope;^39^ uncertainty about the validity, accuracy or precision of estimates^40^ and lack of experience and training in communication skills.^41^

A better understanding is needed of the interaction between survival expectations and behaviour in chronic disease. If compelling evidence is found showing overestimation of survival leads patients to make decisions out of keeping with their likely future, approaches to adjusting such expectations could be developed. Inclusion of validated methods for estimating and communicating prognosis in decision support materials may be one way of increasing the frequency of prognostic discussions. Research into the acceptability and best methodology for facilitating these discussions should be a research priority. Some patients will not feel able to discuss prognosis, so clinicians must take care to elucidate preferences for information. However, clinicians should continue to provide opportunities for prognostic discussion, since preferences may change over time and with disease progression. In other diseases such as breast cancer, the use of prognostic models and decision tools has been shown to increase understanding of prognosis and treatment options, leading to higher degrees of satisfaction.^42^ Validated tools to help predict survival in chronic disease are available,^23^ ^43–45^ but there is no evidence that these are widely employed. Only a minority are provided with accessible calculators (box 1). Studies are needed to examine how prognostic tools can be used in the clinical setting.^46^ It is possible that clinical practice has not kept pace with the paradigm shift towards information sharing with patients. Even where prognostic discussions happen, survival statistics may be misrepresented or censored.^47^ In one study included in this review, nephrologists provided estimates of life expectancy for 89% of the interviewed patients, but reported they would withhold over half of these estimates in clinical practice.^20^

Box 1 **Online calculators available for predicting survival in chronic disease**

The BODE index: 4-year survival in COPD

- http://www.qxmd.com/calculate-online/respirology/bode-index

The Seattle Heart Failure Model: 1, 2 and 3-year survival in HF

- https://depts.washington.edu/shfm/

Integrated Prognostic Model: 6-month mortality on haemodialysis

- http://www.qxmd.com/calculate-online/nephrology/predicting-6-month-mortality-on-hemodialysis

The ability to make firm conclusions from the literature was highly limited by the lack of available evidence. The literature comes largely from single centre cohorts and is of medium to low quality. Data from diseases other than HF is extremely limited, and those with the most advanced disease were under-represented. Included studies are likely to have come from centres where prognostication is considered important. We excluded studies including only participants with cancer, HIV/AIDS, congenital heart disease, cystic fibrosis and organ transplant. Cancer literature has been well summarised,^3^ but it is possible that these excluded conditions could have provided additional insight. We are aware of only one paper that would have been included without this exclusion, showing that young adults with congenital heart disease expect to live almost as long as their healthy peers.^48^

There is no standardised or validated method for assessing self-estimated life expectancy, and it is likely that responses are influenced by methodology. Additionally, asking a patient how long they expect to live facilitates a quantitative assessment of their understanding but does not provide information on how such perceptions are formed and influenced. Large numbers of patients were excluded from the studies or were unable or unwilling to estimate their own life expectancy, with the potential to introduce bias. In addition, many patients were excluded on grounds of language skills or cognitive impairment. These excluded individuals are likely to find discussing and understanding prognosis particularly challenging, and this undermines the relevance of the included studies to a population of patients with chronic disease, in whom cognitive impairment is common. All the studies reporting actual survival were limited by short follow-up times and low numbers of deaths in the cohorts. Hospitalised patients were under-represented in the included studies. It is feasible that survival expectations are different during periods of acute illness requiring admission; the point at which critical decisions about healthcare are often made. There is evidence to suggest that overestimation of survival persists in these situations however; in malignant and non-malignant disease.^2^ ^32^ ^34^ ^49^

None of the included studies had a healthy reference group. Overestimation of life expectancy cannot, therefore, be presumed a phenomenon limited to patients with disease. A recently published prospective cohort study provides some evidence to suggest self-estimation of survival might be different among individuals unselected for chronic disease. Approximately half of participants made predictions of their life expectancy consistent with those from a statistical model.^50^ Where predictions were inaccurate, they were approximately three times more likely to be underestimates than overestimates. Overestimation increased with age, but it is unclear whether this represented an independent effect of ageing on subjective life expectancy, or confounding by the increased prevalence of disease. It is possible that general population studies of self-estimated life expectancy could be analysed for differences between individuals with and without disease.

## Conclusions

Patients with non-cancer chronic disease may have survival expectations that markedly exceed outcomes. These expectations might lead some patients to make health decisions and life choices that they would not if their predictions were more realistic. A better understanding is needed of the interaction between survival expectations and behaviour in chronic disease. If compelling evidence is found showing overestimation of survival leads patients to make decisions out of keeping with their likely future, approaches to adjusting such expectations could be developed. Meanwhile, clinicians caring for patients with chronic disease must make attempts to elucidate what prognostic information each patient already knows, wants to know and might benefit from knowing. Appropriate information should then be shared in a form that the patient can use to inform their decisions.
