# Supplementary material for: How long do patients with chronic disease expect to live? A systematic review of the literature
Source: BMJ Open. 2016 Dec 21;6(12):e012248. doi: 10.1136/bmjopen-2016-012248 (PMC5223727; doi:10.1136/bmjopen-2016-012248)
Supplement: supplementary appendix [file bmjopen-2016-012248supp_appendixA.pdf]

Search plan

Combining terms for 'life expectancy' AND 'self-estimated'

- Medline 1946, Embase (including Cochrane) 1974, PsycINFO 1806 to present day (date of search 3rd October 2016)
- Limited to English, humans
- Fingertip search of the reference lists of all included papers and reviews on the subject

Terms for 'life expectancy'

Mesh

Exp Prognosis

Exp Life expectancy

Text word

Prognosis.ti,ab

Life expect$.ti,ab

Life duration.ti,ab

Length of life.ti,ab

Duration of life.ti,ab

Days left.ti,ab

Weeks left.ti,ab

Months left.ti,ab

Years left.ti,ab

Survival benefit.ti,ab

Life left.ti,ab

Period of existence.ti,ab

Long term survival.ti,ab

Short term survival.ti,ab

Medium term survival.ti,ab

Life exten$.ti,ab

Prognos$ expect$.ti,ab

Predict$ surviv$.ti,ab

"Within 5"

(Chance$ adj5 surviv$).ti,ab

(Expect$ adj5 alive).ti,ab

(Surviv$ adj5 Estimat$).ti,ab

(Surviv$ adj5 probab$).ti,ab

(Surviv$ adj5 expect$).ti,ab

(Surviv$ adj5 Predict$).ti,ab

(Estimat$ adj5 prognosis).ti,ab

(Prognos$ adj5 expect$).ti,ab

Terms for 'self-estimated'

Text word

Patient$ estimat$.ti,ab

Self estimat$.ti,ab

Patient$ predict$.ti,ab

Patient expect$.ti,ab

Self assess$.ti,ab

Self forcast$.ti,ab

Self generate$.ti,ab

Self estimate$.ti,ab

Patient$ generat$.ti,ab

Patient$ forcast$.ti,ab

Personal$ estimat$.ti,ab

Personal$ forecast$.ti,ab

Prognos$ belie$.ti,ab

(Prognos$ adj5 disclos$).mp.

(Perceiv$ adj5 prognos$).mp.

(Communicat$ adj5 prognos$).mp.

(Understand$ adj5 prognos$).mp.

"Within 5"

(Own adj3 estimat$).ti,ab

Grey literature:

The grey literature was searched using ProQuest dissertations and theses search and the Networked Digital Library of Theses and Dissertations Global ETD search. Databases were searched for English language manuscripts where the abstract contained the terms "life expectancy" and "perceived" or "self-estimated".
