# Supplementary material for: How long do patients with chronic disease expect to live? A systematic review of the literature
Source: BMJ Open. 2016 Dec 21;6(12):e012248. doi: 10.1136/bmjopen-2016-012248 (PMC5223727; doi:10.1136/bmjopen-2016-012248)
Supplement: supplementary appendix [file bmjopen-2016-012248supp_appendixB.pdf]

Quality assessment tool:

1) Was the sample representative of patients in the general population with chronic life-limiting non-cancer disease?
    a) Truly representative
    b) Somewhat representative
    c) Poorly representative or insufficient description of the of the group provided

2) Was the method by which the sample was identified, recruited and retained described?
    a) Clear description/diagram illustrating recruitment, consent, exclusion, loss to follow up, death etc.
    b) Unclear or incomplete description/diagram
    c) Poor or no description of process provided

3) Were biases generated by the selection process; for example due to a very low participation rate, an all-volunteer sample or extremely restricted sampling?
    a) Selection bias unlikely
    b) Selection bias possible
    c) Selection bias very likely

4) Was a control or comparison group available?
    a) A well matched control/comparison group was available
    b) A poorly matched control/comparison group was available
    c) No control/comparison group was available

5) Were the measures used well-chosen to provide a serviceable assessment of self-estimation of life-expectancy?
    a) Measures likely to provide a high quality assessment of self-estimated life expectancy
    b) Measures moderately likely to provide a high quality assessment of self-estimated life expectancy
    c) Measures unlikely to provide a high quality assessment of self-estimated life expectancy

6) Is comparator data available to provide a test of the accuracy of the patient’s estimate?
    a) Prospective collection of actual survival statistics
    b) Use of physician estimates, predictive models, or equivalent
    c) Disease standard survival only, or no comparator data used

Result:

For each question, A = 3, B = 2, C = 1. Mean score from reviewers. 6-9 = Low quality, 10-14= medium quality, 15-18 = high quality
