# Supplementary material for: How long do patients with chronic disease expect to live? A systematic review of the literature
Source: BMJ Open. 2016 Dec 21;6(12):e012248. doi: 10.1136/bmjopen-2016-012248 (PMC5223727; doi:10.1136/bmjopen-2016-012248)
Supplement: supplementary appendix [file bmjopen-2016-012248supp_appendixC.pdf]

Appendix C: Complete list of full papers considered

Author/Date	Title	Journal	Accepted/Rejected	Reasoning
**(Allen et al. 2008)**	Discordance between patient-predicted and model-predicted life expectancy among ambulatory patients with heart failure	Journal of the American Medical Association	**Accepted**	**Meets criteria:** Patients with heart failure were asked how long they expect to live.
**(Ambardekar et al. 2016)**	Conflicting Perceptions of Prognosis and Treatment Options between Physicians and Patients with Advanced Heart Failure: Results From the Medical Arm of Mechanically Assisted Circulatory Support (Medamacs) Registry	Journal of Cardiac Failure	**Accepted, abstract only**	**Meets criteria:** Patients with heart failure were asked how long they expect to live.
**(Belkora et al. 2011)**	Does use of the adjuvant! Model influence use of adjuvant therapy through better risk communication?	Journal of the National Comprehensive Cancer Network	Rejected	Patients with cancer only
**(Brouwer and van Exel 2005)**	Expectations regarding length and health related quality of life: some empirical findings	Social science and medicine	Rejected	Questionnaire applied to members of public, rather than individuals with chronic disease
**(Chen et al. 2013)**	Expectations about the effectiveness of radiation therapy among patients with incurable lung cancer	Journal of Clinical Oncology	Rejected	Patients with cancer only
**(Christakis and Lamont 2000)**	Extent and determinants of error in doctors' prognoses	British Medical Journal	Rejected	Doctors, but not patients predicted life-expectancy

	in terminally ill patients: Prospective cohort study			
**(Connors 1995)**	A Controlled Trial to Improve Care for Seriously Ill Hospitalized Patients	Journal of the American Medical Association	Rejected	Patients only asked about the likelihood of being alive at two months
**(Edwards and Baharani 2015)**	Beyond Believing- Thoughts on End of Life from Haemodialysis Patients at the End of Life	Nephrology Dialysis Transplantation	Rejected	No quantitative data for self-estimated life expectancy made
**(Enzinger et al. 2013)**	Outcomes of prognostic disclosure: Effects on advanced cancer patients' prognostic understanding, mental health, and relationship with their oncologist	Journal of Clinical Oncology	Rejected	Patients with cancer only
**(Fisher et al. 2015)**	Patient characteristics associated with prognostic awareness: a study of a Canadian palliative care population using the InterRAI palliative care instrument	Journal of Pain and Symptom Management	Rejected	Whilst study reports on awareness of six month prognosis patients were not asked directly to estimate their life expectancy. Data gathered from interviewer subjective inference.
**(Fried, Bradley, and O'Leary 2003)**	Prognosis Communication in Serious Illness: Perceptions of Older Patients, Caregivers, and Clinicians	Journal of the American Geriatrics Society	**Accepted**	**Meets criteria:** Patients with advanced heart failure, COPD and cancer asked how long they expect to live.<br><br>Authors provided additional data to permit analysis of non-cancer diagnoses alone.

**(Fried, Bradley, and O'Leary 2006)**	Changes in prognostic awareness among seriously ill older persons and their caregivers	Journal of Palliative Medicine	**Accepted**	**Meets criteria:** Same cohort as 2003 paper, interviewed sequentially.<br><br>Authors provided additional data to permit analysis of non-cancer diagnoses alone.
**(Gleason et al. 2009)**	The influence of patient expectations regarding cure on treatment decisions	Patient Education & Counselling	Rejected	Patients with cancer only.
**(Griffin, Loh, and Hesketh 2013)**	A mental model of factors associated with subjective life expectancy	Social science and medicine	Rejected	Questionnaire applied to unselected members of the public, rather than individuals with chronic disease
**(Gwilliam et al. 2013)**	Prognosticating in patients with advanced cancer-observational study comparing the accuracy of clinicians' and patients' estimates of survival	Annals of Oncology	Rejected	Patients with cancer only
**(Haidet et al. 1998)**	Outcomes, preferences for resuscitation, and physician-patient communication among patients with metastatic colorectal cancer	American Journal of Medicine	Rejected	Patients with cancer only
**(Kitko and Hupcey 2015)**	Patients perceptions of illness severity in advanced heart failure	Heart Failure 2015 and the 2nd World Congress on Acute Heart Failure Seville Spain.	Rejected	Qualitative evidence only
**(Kraai et al. 2013)**	Preferences of heart failure patients in daily clinical practice: Quality of life or	European Journal of Heart Failure	**Accepted**	**Meets criteria:** Patients with advanced heart failure were asked to estimate their own

	longevity?			life expectancy.
**(Krumholz et al. 1998)**	Resuscitation Preferences Among Patients With Severe Congestive Heart Failure : Results From the SUPPORT Project	Circulation	Rejected	Patients only asked about the likelihood of being alive at two months
**(Le Blanc et al. 2014)**	Acute myeloid leukemia (AML) patients' understanding of prognosis and treatment goals: A mixed-methods study	Journal of Clinical Oncology	Rejected	Patients with cancer only
**(Lee et al. 2001)**	Discrepancies between patient and physician estimates for the success of stem cell transplantation	Journal of the American Medical Association	Rejected	Patients with cancer only
**(Lipkus et al. 2010)**	Breast cancer patients' treatment expectations after exposure to the decision aid program adjuvant online: the influence of numeracy	Medical decision making : an international journal of the Society for Medical Decision Making	Rejected	Patients with cancer only
**(Lynn et al. 2000)**	Living and dying with chronic obstructive pulmonary disease	Journal of the American Geriatrics Society	Rejected	Patients only asked about the likelihood of being alive at two months
**(O'Donnell et al. 2015)**	Need to Elicit Patient Preferences for Information About Limited Prognosis in Heart Failure	Journal of Cardiac Failure	**Accepted, abstract only**	**Meets criteria:** Patients with advanced heart failure were asked to estimate how long they expect to live
**(O'Donnell et al. 2003)**	Preferences for cardiopulmonary resuscitation among patients 80 years or older: The views of patients and their	Journal of the American Medical Directors Association	Rejected	Patients only asked about the likelihood of being alive at two months

	physicians			
**(Phillips et al. 1996)**	Choices of seriously ill patients about cardiopulmonary resuscitation: Correlates and outcomes	American Journal of Medicine	Rejected – note multiple sub-studies of the SUPPORT study were rejected during title/abstract searching phase	In the SUPPORT study patients were only asked about the likelihood of being alive at two months
**(Reid et al. 2006)**	Estimates of Life Expectancy by Adolescents and Young Adults With Congenital Heart Disease	Journal of the American College of Cardiology	Rejected	Patients with congenital disease only
**(Sanchez-Menegay and Stalder 1994)**	Do physicians take into account patients' expectations?	Journal of General Internal Medicine	Rejected	No quantitative assessment made of subjective life expectancy
**(Schell et al. 2012)**	Discussions of the kidney disease trajectory by elderly patients and nephrologists: a qualitative study	American Journal of Kidney Disease	Rejected	No quantitative assessment made of subjective life expectancy
**(Sekeres et al. 2004)**	Decision-making and quality of life in older adults with acute myeloid leukemia or advanced myelodysplastic syndrome	Leukemia	Rejected	Patients with cancer only
**(Shah et al. 2006)**	Estimating needs in life threatening illness: A feasibility study to assess the views of patients and doctors	Palliative medicine	**Accepted**	**Meets criteria:** Patients with advanced chronic disease and cancer asked to estimate their life expectancy. Data reported separately.
**(Sheldon, Fetting, and Siminoff 1993)**	Offering the option of randomized clinical trials to cancer patients who overestimate their prognoses with standard	Cancer Investigation	Rejected	Patients with cancer only

	therapies			
**(Siegel, Bradley, and Kasl 2003)**	Self-Rated Life Expectancy as a Predictor of Mortality: Evidence from the HRS and AHEAD Surveys	Gerontology	Rejected	Questionnaire applied to unselected members of public, rather than individuals with chronic disease
**(Stewart et al. 2010)**	Patient expectations from implantable defibrillators to prevent death in heart failure	Journal of Cardiac Failure	**Accepted**	**Meets criteria:** Patients with advanced heart failure asked to estimate their life expectancy.
**(Van Der Wal et al. 2016)**	Heart failure patients' future expectations and their association with disease severity, quality of life, depressive symptoms and clinical outcomes	International Journal of Clinical Practice	Rejected	Qualitative data only – patients were not directly asked to quantitatively estimate their own survival
**(Wachterman et al. 2013)**	Relationship between the prognostic expectations of seriously ill patients undergoing hemodialysis and their nephrologists	Journal of the American Medical Association	**Accepted**	**Meets criteria:** Patients receiving haemodialysis asked to estimate their life expectancy.
**(Weeks et al. 1998)**	Relationship between cancer patients' predictions of prognosis and their treatment preferences.	Journal of the American Medical Association	Rejected	Patients with cancer only

Allen, L. A., J. E. Yager, M. J. Funk, W. C. Levy, J. A. Tulsky, M. T. Bowers, G. C. Dodson, C. M. O'Connor, and G. M. Felker. 2008. 'Discordance between patient-predicted and model-predicted life expectancy among ambulatory patients with heart failure', *JAMA*, 299: 2533-42.

Ambardekar, A. V., J. T. Thibodeau, A. D. DeVore, M. M. Kittleson, R. C. Forde-McLean, M. Palardy, M. M. Mountis, L. Cadaret, J. J. Teuteberg, S. V. Pamboukian, L. W. Stevenson, R. B. Xie, and G. C. Stewart. 2016. 'Conflicting Perceptions of Prognosis and Treatment Options between Physicians

and Patients with Advanced Heart Failure: Results From the Medical Arm of Mechanically Assisted Circulatory Support (Medamacs) Registry', *Journal of Cardiac Failure*, 22: S18-S18.

Belkora, J. K., D. W. Hutton, D. H. Moore, and L. A. Siminoff. 2011. 'Does use of the adjuvant! Model influence use of adjuvant therapy through better risk communication?', *JNCCN Journal of the National Comprehensive Cancer Network*, 9: 707-12.

Brouwer, W. B., and N. J. van Exel. 2005. 'Expectations regarding length and health related quality of life: some empirical findings', *Soc Sci Med*, 61: 1083-94.

Chen, A. B., A. Cronin, J. C. Weeks, E. A. Chrischilles, J. Malin, J. A. Hayman, and D. Schrag. 2013. 'Expectations about the effectiveness of radiation therapy among patients with incurable lung cancer', *Journal of Clinical Oncology*, 31: 2730-5.

Christakis, N. A., and E. B. Lamont. 2000. 'Extent and determinants of error in doctors' prognoses in terminally ill patients: Prospective cohort study', *British Medical Journal*, 320: 469-72.

Connors, Alfred F. 1995. 'A Controlled Trial to Improve Care for Seriously III Hospitalized Patients', *JAMA*, 274: 1591.

Edwards, S., and J. Baharani. 2015. 'Beyond Believing- Thoughts on End of Life from Haemodialysis Patients at the End of Life', *Nephrology Dialysis Transplantation*, 30: 52nd ERA-EDTA Congress. London United Kingdom. Conference Start: 20150528. Conference End: 31. Conference Publication: (var.pagings). 30 (pp iii628).

Enzinger, A. C., B. H. Zhang, T. A. Balboni, D. Schrag, and H. G. Prigerson. 2013. 'Outcomes of prognostic disclosure: Effects on advanced cancer patients' prognostic understanding, mental health, and relationship with their oncologist', *Journal of Clinical Oncology*, 31.

Fisher, K., H. Seow, J. Cohen, A. Declercq, S. Freeman, and D. M. Guthrie. 2015. 'Patient characteristics associated with prognostic awareness: a study of a Canadian palliative care population using the InterRAI palliative care instrument', *J Pain Symptom Manage*, 49: 716-25.

Fried, T. R., E. H. Bradley, and J. O'Leary. 2006. 'Changes in prognostic awareness among seriously ill older persons and their caregivers', *Journal of Palliative Medicine*, 9: 61-69.

Fried, Terri R., Elizabeth H. Bradley, and John O'Leary. 2003. 'Prognosis Communication in Serious Illness: Perceptions of Older Patients, Caregivers, and Clinicians', *J Am Geriatr Soc*, 51: 1398-403.

Gleason, M. E., F. W. Harper, S. Eggly, J. C. Ruckdeschel, and T. L. Albrecht. 2009. 'The influence of patient expectations regarding cure on treatment decisions', *Patient Education & Counseling*, 75: 263-9.

Griffin, Barbara, Vanessa Loh, and Beryl Hesketh. 2013. 'A mental model of factors associated with subjective life expectancy. [References]', *Social Science & Medicine*, 82: 79-86.

Gwilliam, B., V. Keeley, C. Todd, C. Roberts, M. Gittins, L. Kelly, S. Barclay, and P. Stone. 2013. 'Prognosticating in patients with advanced cancer-observational study comparing the accuracy of clinicians' and patients' estimates of survival', *Annals of Oncology*, 24: 482-88.

Haidet, P., M. B. Hamel, R. B. Davis, N. Wenger, D. Reding, P. S. Kussin, A. F. Connors, J. Lynn, J. C. Weeks, R. S. Phillips, and SUPPORT Investigators. 1998. 'Outcomes, preferences for resuscitation, and physician-patient communication among patients with metastatic colorectal cancer', *American Journal of Medicine*, 105: 222-29.

Kitko, L., and J. Hupcey. 2015. 'Patients perceptions of illness severity in advanced heart failure', *European Journal of Heart Failure*, Conference: Heart Failure 2015 and the 2nd World Congress on Acute Heart Failure Seville Spain. Conference Start: 20150523 Conference End: 26. Conference Publication: (var.pagings). 17 (pp 139).

Kraai, I. H., K. M. Vermeulen, M. L. A. Luttik, T. Hoekstra, T. Jaarsma, and H. L. Hillege. 2013. 'Preferences of heart failure patients in daily clinical practice: Quality of life or longevity?', *European Journal of Heart Failure*, 15: 1113-21.

Krumholz, H. M., R. S. Phillips, M. B. Hamel, J. M. Teno, P. Bellamy, S. K. Broste, R. M. Califf, H. Vidaillet, R. B. Davis, L. H. Muhlbaier, A. F. Connors, J. Lynn, and L. Goldman. 1998. 'Resuscitation Preferences Among Patients With Severe Congestive Heart Failure : Results From the SUPPORT Project', *Circulation*, 98: 648-55.

Le Blanc, T. W., C. T. Bloom, D. M. Davis, S. C. Locke, K. E. Steinhauser, P. A. Ubel, J. A. Tulsky, and A. P. Abernethy. 2014. 'Acute myeloid leukemia (AML) patients' understanding of prognosis and treatment goals: A mixed-methods study', *Journal of Clinical Oncology*, 1).

Lee, S. J., D. Fairclough, J. H. Antin, and J. C. Weeks. 2001. 'Discrepancies between patient and physician estimates for the success of stem cell transplantation', *Journal of the American Medical Association*, 285: 1034-38.

Lipkus, Isaac M., Ellen Peters, Gretchen Kimmick, Vlayka Liotcheva, and Paul Marcom. 2010. 'Breast cancer patients' treatment expectations after exposure to the decision aid program Adjuvant Online: The influence of numeracy. [References]', *Medical Decision Making*, 30: 464-73.

Lynn, J., E. W. Ely, Z. Zhong, K. L. McNiff, N. V. Dawson, A. Connors, N. A. Desbiens, M. Claessens, and E. P. McCarthy. 2000. 'Living and dying with chronic obstructive pulmonary disease', *J Am Geriatr Soc*, 48: S91-S100.

O'Donnell, A., K. Schaefer, M. Young, K. Walsh, A. Porter, L. Stevenson, and A. Desai. 2015. 'Need to Elicit Patient Preferences for Information About Limited Prognosis in Heart Failure', *Journal of Cardiac Failure*, 21: S63-S64.

O'Donnell, H., R. S. Phillips, N. Wenger, J. Teno, R. B. Davis, and M. B. Hamel. 2003. 'Preferences for cardiopulmonary resuscitation among patients 80 years or older: The views of patients and their physicians', *Journal of the American Medical Directors Association*, 4: 139-44.

Phillips, R. S., N. S. Wenger, J. Teno, R. K. Oye, R. Califf, P. Layde, N. Desbiens, A. F. Connors, and J. Lynn. 1996. 'Choices of seriously ill patients about cardiopulmonary resuscitation: Correlates and outcomes', *American Journal of Medicine*, 100: 128-37.

Reid, G. J., G. D. Webb, M. Barzel, B. W. McCrindle, M. J. Irvine, and S. C. Siu. 2006. 'Estimates of Life Expectancy by Adolescents and Young Adults With Congenital Heart Disease', *Journal of the American College of Cardiology*, 48: 349-55.

Sanchez-Menegay, C., and H. Stalder. 1994. 'Do physicians take into account patients' expectations?', *Journal of General Internal Medicine*, 9: 404-06.

Schell, J. O., U. D. Patel, K. E. Steinhauser, N. Ammarell, and J. A. Tulsky. 2012. 'Discussions of the kidney disease trajectory by elderly patients and nephrologists: a qualitative study', *Am J Kidney Dis*, 59: 495-503.

Sekeres, M. A., R. M. Stone, D. Zahrieh, D. Neuberg, V. Morrison, D. J. De Angelo, I. Galinsky, and S. J. Lee. 2004. 'Decision-making and quality of life in older adults with acute myeloid leukemia or advanced myelodysplastic syndrome', *Leukemia*, 18: 809-16.

Shah, S., M. Blanchard, A. Tookman, L. Jones, R. Blizard, and M. King. 2006. 'Estimating needs in life threatening illness: A feasibility study to assess the views of patients and doctors', *Palliative Medicine*, 20: 205-10.

Sheldon, J. M., J. H. Fetting, and L. A. Siminoff. 1993. 'Offering the option of randomized clinical trials to cancer patients who overestimate their prognoses with standard therapies', *Cancer Investigation*, 11: 57-62.

Siegel, Michele, Elizabeth H. Bradley, and Stanislav V. Kasl. 2003. 'Self-Rated Life Expectancy as a Predictor of Mortality: Evidence from the HRS and AHEAD Surveys', *Gerontology*, 49: 265-71.

Stewart, G. C., J. R. Weintraub, P. P. Pratibhu, M. J. Semigran, J. M. Camuso, K. Brooks, S. W. Tsang, M. S. Anello, V. T. Nguyen, E. F. Lewis, A. Nohria, A. S. Desai, M. M. Givertz, and L. W. Stevenson. 2010. 'Patient Expectations From Implantable Defibrillators to Prevent Death in Heart Failure', *Journal of Cardiac Failure*, 16: 106-13.

Van Der Wal, M. H. L., A. Stromberg, D. J. Van Veldhuisen, and T. Jaarsma. 2016. 'Heart failure patients' future expectations and their association with disease severity, quality of life, depressive symptoms and clinical outcomes', *International Journal of Clinical Practice*, 70: 469-76.

Wachterman, M. W., E. R. Marcantonio, R. B. Davis, R. A. Cohen, S. S. Waikar, R. S. Phillips, and E. P. McCarthy. 2013. 'Relationship between the prognostic expectations of seriously ill patients undergoing hemodialysis and their nephrologists', *JAMA Intern Med*, 173: 1206-14.

Weeks, J. C., E. F. Cook, S. J. O'Day, L. M. Peterson, N. Wenger, D. Reding, F. E. Harrell, P. Kussin, N. V. Dawson, A. F. Connors Jr, J. Lynn, and R. S. Phillips. 1998. 'Relationship between cancer patients' predictions of prognosis and their treatment preferences', *Journal of the American Medical Association*, 279: 1709-14.
